# Advancing Target Identification of Nitrated Phospholipids in Biological Systems by HCD Specific Fragmentation Fingerprinting in Orbitrap Platforms

**DOI:** 10.3390/molecules25092120

**Published:** 2020-05-01

**Authors:** Bruna Neves, Sofia Duarte, Pedro Domingues, Dolores Pérez-Sala, Maria Manuel Oliveira, Maria do Rosário Domingues, Tânia Melo

**Affiliations:** 1 Mass Spectrometry Centre, LAQV-REQUIMTE, Department of Chemistry, University of Aveiro, Santiago University Campus, 3810-193 Aveiro, Portugal; brunabneves@ua.pt (B.N.); p.domingues@ua.pt (P.D.); mrd@ua.pt (M.d.R.D.); 2 CESAM—Centre for Environmental and Marine Studies, Department of Chemistry, University of Aveiro, Santiago University Campus, 3810-193 Aveiro, Portugal; 3 Department of Structural and Chemical Biology, Centro de Investigaciones Biológicas Margarita Salas, CSIC, 28040 Madrid, Spain; sofiaineslealduarte@gmail.com (S.D.); dperezsala@cib.csic.es (D.P.-S.); 4 Department of Chemistry, University of Trás-os-Montes e Alto Douro, 5000-801 Vila Real, Portugal; mmso@utad.pt

**Keywords:** lipidomics, phospholipids, nitration, tandem mass spectrometry, higher collision-induced dissociation (HCD)

## Abstract

Nitrated phospholipids have recently been detected *in vitro* and *in vivo* and associated with beneficial health effects. They were identified and quantified in biological samples by lipidomics methodologies using liquid chromatography-collision-induced dissociation (CID) tandem mass spectrometry (MS/MS) acquired with the linear ion trap mass spectrometer. Only a few studies have used higher-energy collision dissociation (HCD)-MS/MS in high-resolution Orbitraps to characterize nitrated phosphatidylserines and nitrated cardiolipins, highlighting the marked differences in the fragmentation patterns when using CID or HCD fragmentation methods. In this study, we aimed to evaluate the fragmentation of nitrated phosphatidylcholine and nitrated phosphatidylethanolamine species under HCD-MS/MS. We studied the effect of normalized collision energy (NCE) in the fragmentation pattern to identify the best acquisition conditions and reporter ions to detect nitrated phospholipids. The results showed that the intensity of the typical neutral loss of nitrous acid (HNO_2_) diminishes with increasing NCE, becoming non-detectable for a higher NCE. Thus, the loss of HNO_2_ could not be the most suitable ion/fragment for the characterization of nitrated phospholipids under HCD. In HCD-MS/MS new fragment ions were identified, corresponding to the nitrated fatty acyl chains, NO_2_-RCOO^−^, (NO_2_-RCOOH-H_2_O + H)^+^, and (NO_2_-RCOOH + H)^+^, suggested as potential reporter ions to detect nitrated phospholipids when using the HCD-MS/MS lipidomics analysis.

## 1. Introduction

Phospholipids (PLs), mainly phosphatidylcholines (PCs) and phosphatidylethanolamines (PEs) are major constituents of cell membranes playing a crucial role in the integrity and function of membranes. PLs can react with reactive oxygen species (ROS) and reactive nitrogen species (RNS), leading to a plethora of structurally different and chemically diverse products. Oxidized PLs have been widely studied [1,2,3], but the modified PLs generated during the reaction with RNS were scarcely addressed. RNS reactions with free fatty acids (FAs) have been described and yielding free nitro FAs (NO_2_-FAs) and nitroxidized FAs [4,5,6] have been recognized as important endogenous signalling molecules [7,8,9]. On the other hand, nitrated PLs have only been reported recently in *in vitro* model systems and biological samples and structurally characterized by mass spectrometry (MS) approaches [10,11,12,13].

Nitrated PCs and nitrated PEs were detected in the cardiac mitochondria of diabetic rats and cardiomyocytes under starvation using liquid chromatography (LC) coupled to a linear ion trap mass spectrometer (LIT) and were characterized by low energy collision-induced dissociation (CID) tandem mass spectrometry (MS/MS) [10,12]. However, nowadays, lipidomics studies are mainly carried out by LC-MS/MS using high-resolution instruments, including orbitrap analysers and higher-energy collisional dissociation (HCD) as fragmentation method [14,15,16,17]. Different fragmentation patterns can be obtained from these two different ion activation methods, CID and HCD, as previously reported for saccharides and peptides [18,19]. In the case of molecular lipid species, it has been described that the combined use of HCD and CID lead to complementary fragmentation patterns [17]. Moreover, a recent study on the characterization of nitrated and nitroxidized derivatives of phosphatidylserine (PS), using CID-LIT and HCD-Orbitrap mass spectrometers, showed dissimilarities in the MS/MS data, related to variations in the relative abundance of typical reporter product ions which have been previously proposed for the identification of nitrated PLs by CID-MS/MS [10,12].

Tandem MS analysis using the HCD or CID fragmentation methods requires the optimization of several instrumental parameters, including collision energy [17]. The collision energy (CE) determines the signal intensity and the type of fragment ions that are generated [17,20,21,22]. The relative abundance of reporter ions and the quality of the MS/MS spectra depend on the applied CE. Hence, the MS/MS spectra acquired under different CEs can be significantly different [20]. Even small differences in the CE used can have a significant impact on the detection of reporter fragment ions [17]. Morever, the optimal CE value for ion activation by HCD and CID may be different [17] because in HCD beam-type collisions occur under a higher pressure than in CID, in particular in ion traps, where dissociation occurs by resonance activation. As such, HCD produces an electron impact (EI)-like fragmentation, with a wider range of fragmentation pathways, and improves the yield of fragment ions at low *m*/*z* [17,23]. Therefore, fine-tuning the CE to achieve optimal fragmentation efficiency is essential to increase the precision and accuracy of the identification of the target compounds.

In some cases, the higher the *m*/*z* value, the higher the CE required for the optimal fragmentation efficiency [24,25]. HCD-MS/MS in orbitrap instruments uses normalized collision energy (NCE) which improves the efficiency of fragmentation over a wide range of *m*/*z* values. In NCE, the collision energy is applied as a function of the *m*/*z* value of the precursor ion. This makes it possible to normalize the differences between the *m*/*z* values and the ideal CE necessary for the optimal activation of the precursor ions, maximizing the structural information which is recovered [17,19].

Lipidomics is nowadays based on the use of MS-based approaches using high-resolution MS instruments such as Orbitraps, with HCD-MS/MS and high resolution quadrupole–time of flight (Q-TOF) with CID-MS/MS, and both MS/MS modes can give dissimilar MS/MS spectra [14,15,16,17]. Thus, it is relevant to study the characteristic HCD-MS/MS fragmentation patterns of the nitrated derivatives of the most abundant PLs, PCs and PEs found in mammalian cells and biofluids. Indeed, the approaches based on MS are the most appropriate for identifying nitrated PLs in biological samples and for revealing their putative physiological roles. This information could provide new clues for the discovery of new biomarkers or therapeutic strategies, as proposed for free nitrated FAs [26,27,28].

Therefore, this study aimed to identify the reliable reporter ions and fragmentation patterns of nitrated and nitroxidized PCs and PEs using lipidomic approaches based on electrospray (ESI) high resolution (HR) HCD-MS/MS performed on a Q-Exactive hybrid quadrupole orbitrap mass spectrometer. The analysis using ESI-HR-HCD-MS/MS experiments was carried out using nitrated PCs and PEs standards generated in an *in vitro* model of nitration, which was validated for the qualitative and quantitative determination of the nitrated PLs in the lipid extracts from human adrenal cortex adenocarcinoma cells (SW13/cl.2 cells) treated with nitrated 1-palmitoyl-2-oleoyl-*sn*-glycero-3-phosphocholine (POPC). In this study, we also evaluated the impact of different NCEs on the relative abundance of the reporter product ions which were used to identify nitrated and nitroxidized PLs in *in vitro* model systems and in the lipid extracts from cells.

## 2. Results

The nitrated and nitroxidized derivatives of PCs and PEs, the two main classes of PLs found in biological samples, were obtained using nitronium tetrafluoroborate (NO_2_BF_4_) in a mimetic model of nitration mediated by RNS which occured in the hydrophobic environment of biological membranes [10,12]. This reaction led to the formation of different PL nitration products with high yields, of which, nitro PL (NO_2_-PL) was one of the main species [10,12]. These modified lipids were analyzed using ESI-MS in the both positive (for PCs and PEs) and negative ionization modes (for PEs). The structural characterization was carried out by ESI-HR-HCD-MS/MS using three different NCE to evaluate the effects on the relative abundance of reporter fragment ions used for the detection of nitrated and nitroxidized PLs. In all the experiments, only the NCE was modified. All the other procedures, namely the preparation of the samples, the additional acquisition parameters in the MS and MS/MS modes and the methods of processing the data, were maintained between acquisitions. The additional MS and MS/MS parameters used in this work, such as gas flow, capillary temperature, automatic gain control, maximum injection time and resolution were selected taking into account the recommendations of the manufacturers during the installations of the instrument but also based on our previous lipidomic studies that allowed us to develop several optimization steps to retrieve the most useful information during the data acquisition. Using this approach, we assessed whether the relative abundance of the different product ions, namely the typical reporter ions and the carboxylate anions of the modified fatty acyl chain, was different between the molecular species and depended on the HCD-MS/MS parameters.

### 2.1. Optimization of the Normalized Collision Energy for the Study of Nitrated PL Standards

PCs and PEs standards carrying different unsaturated fatty acyl chains (OA-oleic acid, LA-linoleic acid and AA-arachidonic acid), namely 1-palmitoyl-2-oleoyl-*sn*-glycero-3-phosphocholine (POPC), 1-palmitoyl-2-linoleoyl-*sn*-glycero-3-phosphocholine (PLPC) and 1-palmitoyl-2-arachidonoyl-*sn*-glycero-3-phosphocholine (PAPC) for the PC class and 1-palmitoyl-2-oleoyl-*sn*-glycero-3-phosphoethanolamine (POPE), 1-palmitoyl-2-linoleoyl-*sn*-glycero-3-phosphoethanolamine (PLPE) and 1-palmitoyl-2-arachidonoyl-*sn*-glycero-3-phosphoethanolamine (PAPE) for the PE class were nitrated and analyzed. They are generally among the most abundant PLs in cells [10,12]. The nitrated PC and PE species generated using an *in vitro* model system were identified by ESI-MS and the fragmentation obtained under the ESI-HR-HCD-MS/MS was analyzed. Modified PCs were analyzed in positive ion mode ((M + H)^+^ ions), while modified PEs were analyzed in positive ((M + H)^+^ ions) and negative ion modes ((M − H)^−^ ions). All the data were acquired using different concentrations (1 µg mL^−1^, 2 µg mL^−1^ and 4 µg mL^−1^) of the nitrated/nitroxidized PL derivatives. The identification was confirmed by accurate mass measurement (error < 5 ppm) and the elemental composition from the HR-MS data (Tables S1–S9). The confirmation of structural features was obtained by the data analysis of the HCD-MS/MS experiments.

**Figure 1:**
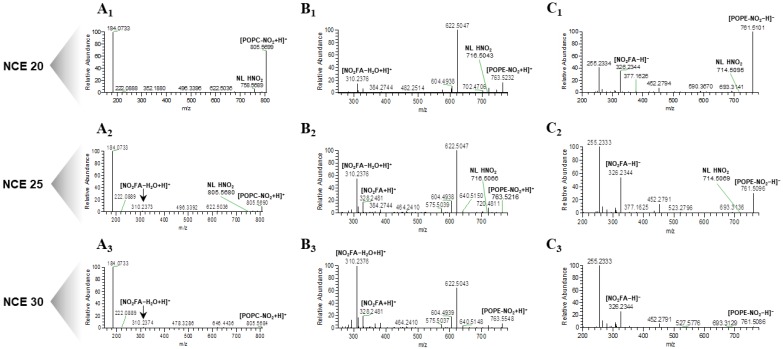
Electrospray (ESI)-high resolution (HR)-HCD-MS/MS spectra of the ions (**A**) (NO_2_-POPC + H)^+^ at *m*/*z* 805.6, (**B**) (NO_2_-POPE + H)^+^ at *m*/*z* 763.5 and (**C**) (NO_2_-POPE − H)^−^ ions at, *m*/*z* 761.5, respectively, acquired in a Q-Exactive Orbitrap mass spectrometer at a concentration of 2 µg mL^−1^, with a low normalized collision energy (NCE) (20) **A1**, **B1**, **C1**; medium NCE (25) **A2**, **B2**, **C2** and high NCE (30) **A3**, **B3**, **C3**, respectively.

The ESI-HR-HCD-MS/MS data obtained in the Q-Exactive Orbitrap for the nitro derivatives of POPC (NO_2_-POPC) and POPE (NO_2_-POPE) are presented in Figure 1. The two species were previously detected *in vivo* [10,12] and nitro OA (NO_2_-OA) was the most abundant NO_2_-FA found in human plasma [29,30]. The HCD-MS/MS spectrum of each nitro PL was acquired using three different NCE: low (20), medium (25), and high (30).

**Figure 2:**
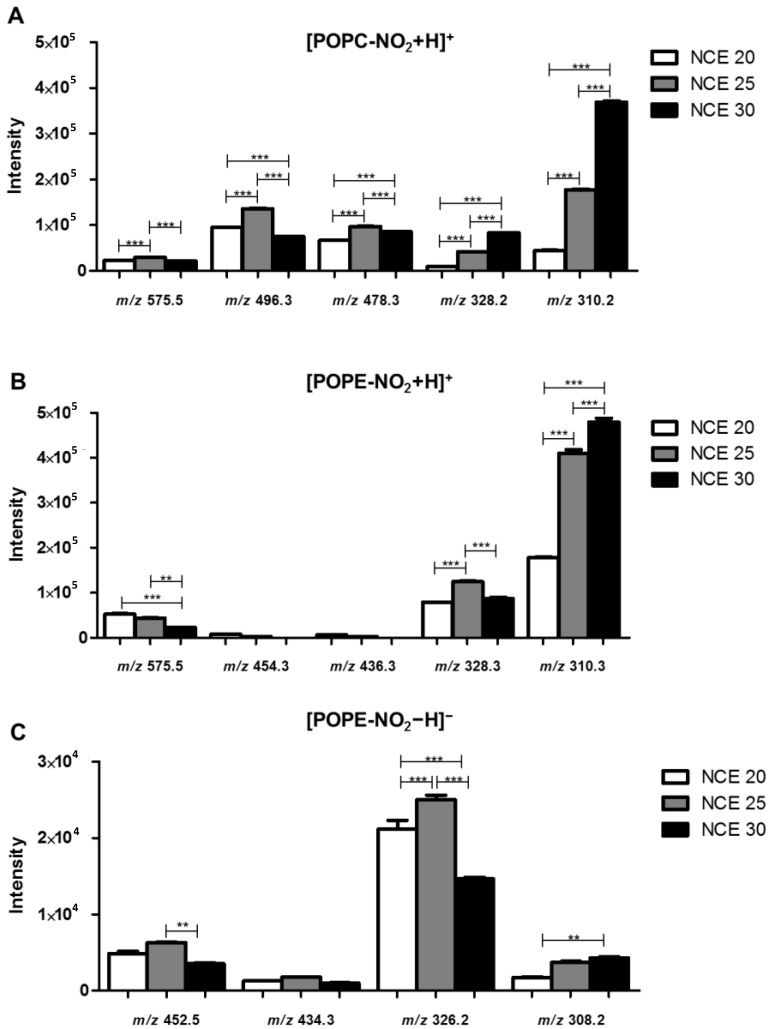
Effect of the low (20), medium (25) and the high (30) NCE in the intensity of the fragment ions with lower *m*/*z* values identified in the HCD-MS/MS spectra of: (**A**) NO_2_-POPC ((M + H)^+^); (**B**) NO_2_-POPE ((M + H)^+^); (**C**) NO_2_-POPE ((M − H)^−^). The identification of the fragment ions is reported in Table 1. Fragment ions at *m*/*z* 575.5 are formed by the combined neutral loss of nitrous acid (HNO_2)_ with the polar heads of phosphocholine (neutral loss (NL) of 47 plus 183 Da) or phosphoethanolamine (NL of 47 plus 141 Da). The fragment ions at *m*/*z* 496.3 and 478.3 (**A**) and *m*/*z* 454.3 and *m*/*z* 436.3 (**B**) arise from the neutral loss of nitrated oleic acid (NO_2_-OA) as keto (NL of 309 Da), (NO_2_-OA-H_2_O) and acid derivatives (NL of 327 Da, NO_2_-OA), respectively. The fragment ions at *m*/*z* 328.2 and 310.2 (**A**,**B**) correspond to the (NO_2_-OA + H)^+^ and (NO_2_-OA − H_2_O + H)^+^ fragment ions, respectively. Fragment ions at *m*/*z* 452.3 and 434.3 (**C**) correspond to the neutral loss of nitrated oleic acid (NO_2_-OA) as keto and acid derivatives, respectively, and the fragment ions at *m*/*z* 326.3 and *m*/*z* 308.3 correspond to the (NO_2_-OA − H)^−^ and (NO_2_-OA − H_2_O − H)^−^ ions, respectively. All the results were obtained using a concentration of 2 µg mL^−1^ of the PL derivatives. Values are the means of three experiments ± standard deviation (SD). Statistical significance was determined using ANOVA and Tukey’s multiple comparison tests (± SD, ** *p* < 0.01, *** *p* < 0.001).

The analysis of the ESI-HR-HCD-MS/MS spectra of the (M + H)^+^ ions of NO_2_-POPC revealed a major product ion at *m*/*z* 184.073, corresponding to the cation of the phosphocholine polar head group ((H_2_PO_4_[CH_2_]_2_N[CH_3_]_3_)^+^), typical of the PCs class (Figure 1A_1_–A_3_ and Figure S1). Besides, in the HCD-MS/MS spectrum acquired with NCE = 20 (Figure 1A_1_ and Figure S1), it was possible to observe, at *m*/*z* 758.569, the reporter ion which was characteristic of nitrated lipids, formed by the neutral loss (NL) of nitrous acid (HNO_2_, NL of 47 Da). When comparing the MS/MS data obtained with an NCE = 25 (Figure 1A_2_ and Figure S1), the intensity of this typical reporter ion decreased and for NCE = 30 it could not be detected (Figure 1A_3_ and Figure S1). We also observed other product ions at lower *m*/*z* values, corresponding to the protonated molecules of the NO_2_-OA fatty acyl chain, namely (NO_2_-OA + H)^+^ ions, at *m*/*z* 328.248, and the (NO_2_-OA − H_2_O + H)^+^ ions, at *m*/*z* 310.237, respectively (Figure 1A_1_–A_3_, Figure S1). The relative abundance of these ions significantly increased for higher NCE (Figure 2A).

The analysis of the ESI-HR-HCD-MS/MS spectra of NO_2_-POPE in the positive ion mode (Figure 1B_1_–B_3_ and Figure S2) showed the product ion at *m*/*z* 622.504 (C_2_H_6_PO_4_N), formed due to the NL of 141 Da, corresponding to the polar head group of phosphoethanolamine. As observed for NO_2_-POPC, the abundance of the product ions formed due to the typical NL of 47 Da, observed at *m*/*z* 716.504, was also inversely correlated to the increase in the NCE. The product ions of modified FAs were observed at *m*/*z* 328.248 and *m*/*z* 310.238, respectively, namely the (NO_2_-OA + H)^+^ and (NO_2_-OA − H_2_O + H)^+^ ions which were fairly abundant and their abundance increased with NCE. However, for ions at *m*/*z* 328.248 a statistically significant decrease was observed when comparing NCE = 25 and NCE = 30 (Figure 2B).

**Figure 3:**
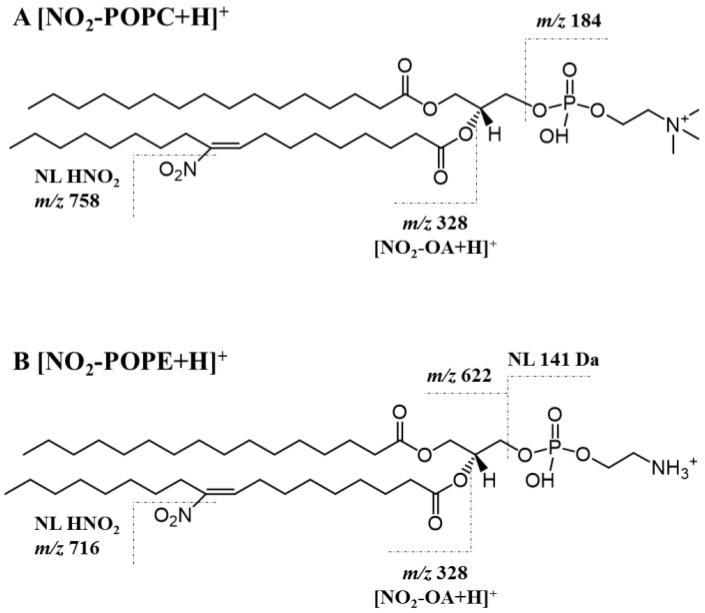
Proposed structure of the (M + H)^+^ ion of nitro POPC (**A**, (NO_2_-POPC + H)^+^) and nitro POPE (**B**, (NO_2_-POPE + H)^+^) with the assignment of the major fragmentation pathways of nitrated phospholipids, namely the neutral loss (NL) of nitrous acid (HNO_2_, NL of 47 Da) at *m*/*z* 758 for NO_2_-POPC and *m*/*z* 716 for NO_2_-POPE, respectively, and the product ions of the modified fatty acyl chain ((NO_2_-OA + H)^+^) at *m*/*z* 328. The typical fragmentation of the polar head group is also assigned: product ions at *m*/*z* 184 for the phosphocholine polar head, and NL of 141 Da for the phosphoethanolamine head group. In these structures the NO_2_ is located in C10, but it is not possible to exclude its location in C9.

In Figure 3 is shown a representative structure of NO_2_-POPC (Figure 3A) and NO_2_-POPE (Figure 3B) with the assignment of the major fragmentation pathways observed in the HCD-MS/MS of the (M + H)^+^ ions of both nitrated species.

Trends similar to those described above were observed in the HCD-MS/MS spectra of the (M – H)^−^ ions of NO_2_-POPE (Figure 1C_1_–C_3_ and Figure S3). In this spectrum, it was possible to detect minor ions arising from the typical NL of HNO_2_, with a relative abundance which was inversely correlated with the increase in NCE and abundant carboxylate anions of nitrated FA at *m*/*z* 326.234 ((NO_2_-OA − H)^−^) namely for NCE = 20 and NCE = 25 (Figure 2C). These product ions at low *m*/*z* value can be proposed as NO_2_-PLs reporter ions, because they were observed with a high relative abundance in the low *m*/*z* range.

In addition, in the mass spectra of PCs and PEs acquired in positive ion mode, we observed the product ions of lower relative abundance, arising from the combined NL of the polar head of PLs and the NL of the nitro group (NO_2_, 47 Da), as well as product ions arising from the NL of the modified fatty acyl chain as acid and ketene derivatives. These ions were also observed in the negative ion mode in the tandem mass spectra of PEs (Figure 2 and Figure S4, and Tables S10 and S11).

The analysis of the HCD-MS/MS spectra acquired for the nitro derivatives of the other PCs and PEs standards (NO_2_-PLPC, NO_2_-PAPC, NO_2_-PLPE and NO_2_-PAPE) revealed the same trend as that described above (Tables S1–S11). In Table 1 are summarized the main fragmentation pathways observed in the HCD-MS/MS spectra of the (M + H)^+^ ions of all the nitrated PLs analysed in this study.

We also studied, using HCD-MS/MS experiments, other nitrated derivatives of PCs and PEs such as dinitro (2NO_2_-PLs, +90 Da) and nitronitroso ((NO_2_)(NO)-PLs, +74 Da) derivatives, as well as with nitroxidized derivatives, such as nitro-hydroxy ((NO_2_)O-PLs, +61 Da) and nitro-hydroperoxy ((NO_2_)2O-PLs, +77 Da), previously characterized by CID-MS/MS [11]. For all of these species, we observed the same tendency described above for NO_2_-POPC and NO_2_-POPE (Tables S1–S11) and the most abundant reporter ions were attributed to the modified fatty acids. The analysis of the HCD-MS/MS data also revealed that the fragmentation pattern was independent of the concentration of the nitrated PL species studied, as shown in Tables S10 and S11.

In conclusion, we can propose that the most suitable reporter ions for the identification of nitrated and nitroxidized PLs strongly depend on the NCE rather than on the modification (nitration and nitroxidation) or the concentration of the nitrated species. Furthermore, the NCE = 25 was, in our instrument, the most suitable for obtaining the proposed range of reporter ions, from the typical NL of HNO_2_ to the product ions of nitrated FAs, with an intensity suitable for being easily detected in the HCD-MS/MS spectra (Figure 1). An NCE = 30 led to the generation of high yields of fragment ions with low *m*/*z* values (Figure 2 and Figure S4), such as the product ions of the nitrated fatty acyl chain. In the HCD-MS/MS spectra acquired at NCE = 20, the product ions formed due to the typical NL of HNO_2_ (47 Da) or those arising from the combined NL of the PLs polar head group and the NL of HNO_2_ could be considered reporter ions; however, for a higher NCE, the product ions corresponding to (_m_NO_n_-FA + O_y_ + H)^+^, (_m_NO_n_-FA + On − H_2_O + H)^+^, (_m_NO_n_-FA + O_y_ − H)^−^ and (_m_NO_n_-FA + O_y_ − H_2_O − H)^−^ (m = 1–2, n = 1–2, y = 0–2) were the most informative. Therefore, it is essential to monitor different fragment ions as potential reporter ions to find the most suitable ones, depending on the NCE selected for the analysis. These reporter ions will be useful for the confident identification of nitrated PLs in untargeted and targeted lipidomic analysis using high-resolution orbitrap instruments.

### 2.2. Identification of Nitrated PL in Cell Lipid Extracts

The new findings have been validated by the identification and quantification of NO_2_-POPC species in the analysis by hydrophilic interaction liquid chromatography (HILIC)-LC-HCD-MS/MS of cellular lipid extracts. For these experiments, SW13/cl.2 adrenal carcinoma cells stably transfected with a green fluorescent protein (GFP)-fusion constructed of the intermediate filament protein vimentin (GFP-vimentin wt) were used [31]. This cellular model was chosen because we recently showed that the treatment of these cells with NO_2_-POPC, but not with POPC, induced a reorganization of GFP-vimentin distribution, thus confirming a biological effect of the nitrated phospholipid [31]. Two experiments were carried out: a) the lipid extracts were obtained from GFP-vimentin SW13/cl.2 cells treated with a solution of nitrated POPC in culture (treated cells); b) the lipid extracts of the untreated cells (control cells) were treated with different volumes of nitrated POPC solution before the acquisition of the LC-MS data (treated extracts). The NO_2_-POPC species that were screened were previously identified *in vitro* and *in vivo* as the most abundant nitrated species [10,12].

**Figure 4:**
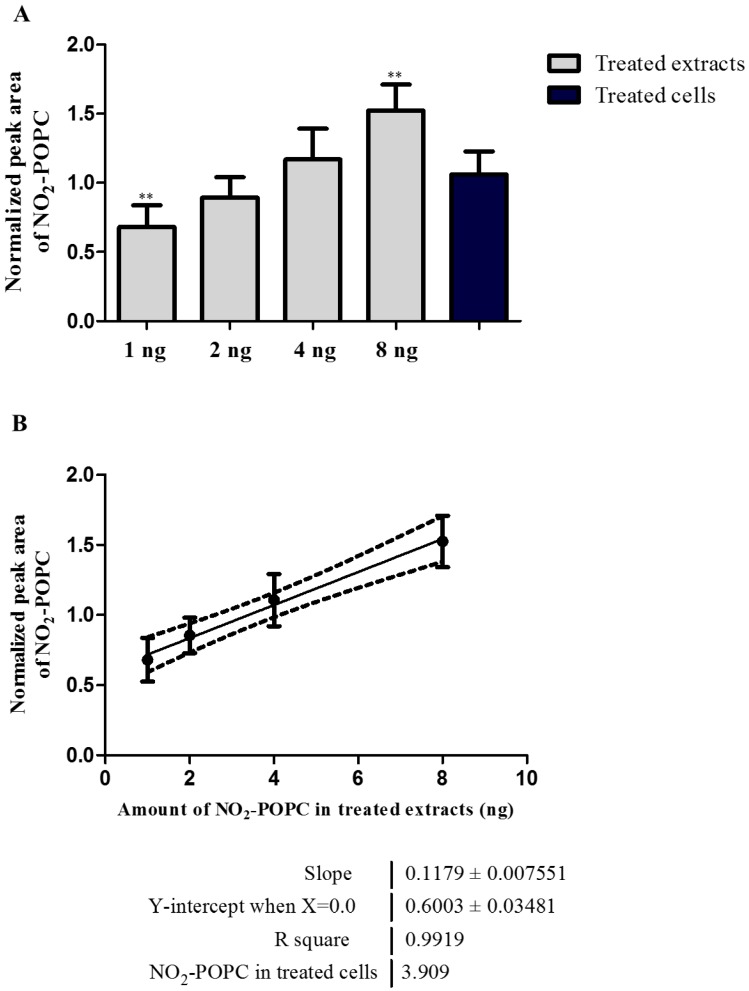
(**A**) NO_2_-POPC at *m*/*z* 805 detected using hydrophilic interaction liquid chromatography (HILIC)- mass spectrometry (MS) and MS/MS analysis in green fluorescent protein (GFP)-vimentin SW13/cl.2 cells treated with 1 µL (1 ng), 2 µL (2 ng), 4 µL (4 ng) and 8 µL (8 ng) of nitrated POPC (1 µg mL^−1^) and the lipid extracts of SW13/cl.2 cells treated with nitrated POPC (10 µmol L^−1^). The results were expressed as peak areas, normalized by the ratio between the peak area of NO_2_-POPC and of the PC internal standard (dMPC, PC 14:0/14:0). The values are the means of six experiments (three biological replicas acquired in duplicate) ± standard deviation (SD). Statistical significance was determined using ANOVA and Tukey’s multiple comparison tests (** *p* < 0.01). (**B**) Linear regression analysis used to estimate the amount of NO_2_-POPC in the lipid extracts from the cells treated with nitrated POPC (10 µmol L^−1^) based on the relationship between the normalized peak area and the amount of NO_2_-POPC in the lipid extracts treated with 1 µL (1 ng), 2 µL (2 ng), 4 µL (4 ng) and 8 µL (8 ng) of nitrated POPC (1 µg mL^−1^).

**Figure 5:**
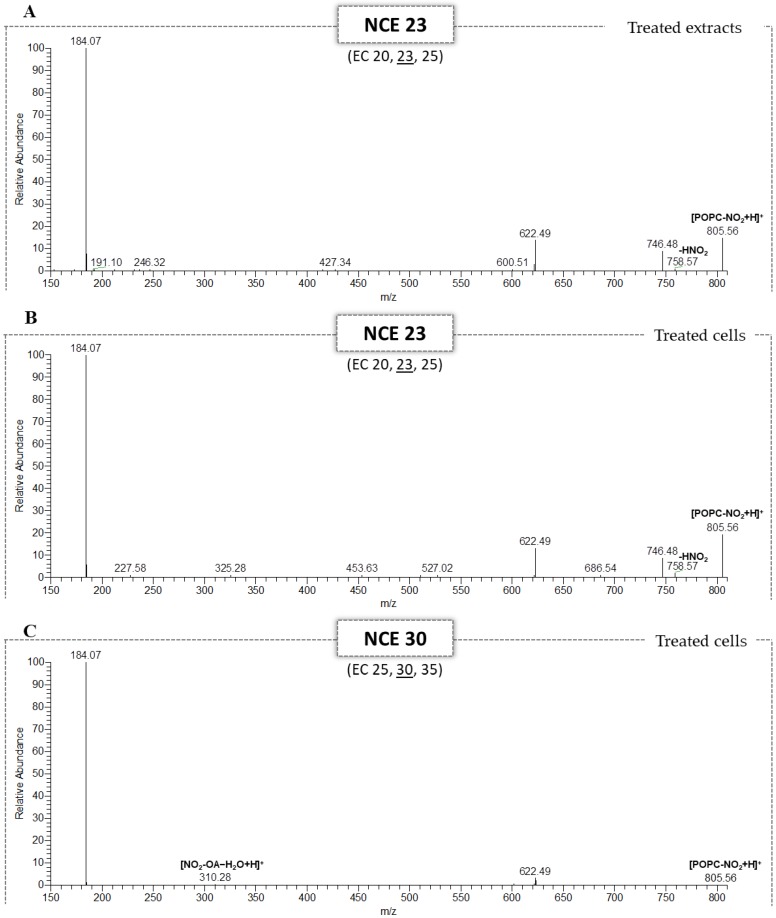
HILIC-HCD-MS/MS spectrum of NO_2_-POPC at *m*/*z* 805.56 identified in the lipid extracts treated with 8 μg of nitrated POPC, obtained with an NCE = 23 (**A**). HILIC-HCD-MS/MS spectra of NO_2_-POPC at *m*/*z* 805.56 of the cellular lipid extracts from the SW13/cl.2 cells treated with nitrated POPC obtained with a stepped NCE range of 20, 23 and 25 (NCE 23) (**B**) and 25, 30 and 35 (NCE 30) (**C**).

The lipid extracts treated with the different amounts of nitrated POPC (1 ng, 2 ng, 4 ng and 8 ng) were analyzed by LC-ESI-HCD-MS/MS using the data-dependent acquisition mode (DDA), as previously described in similar lipidomic studies [14,15]. MS/MS data acquisition was carried out using a stepped NCE analysis which combined the information from the fragment ions obtained at NCEs of 20, 23 and 25 in the same spectrum. NO_2_-POPC identification was based on the retention time (RT) and exact mass measurements (error < 5 ppm), as summarized in Tables S12 and S13. Relative quantification was carried out by integrating the peak area of the extracted ion chromatograms (XIC) of NO_2_-POPC using the MzMine software [31] and an in-house made database containing information for NO_2_-POPC, using a mass tolerance of 5-ppm. The peak area of the NO_2_-POPC species was normalized using the peak area of the PC internal standard (1,2-dimyristoyl-*sn*-glycero-3-phosphocholine, dMPC, PC 14:0/14:0). The results are presented in Figure 4 and Tables S14–S17. As expected, the higher the amount of nitrated POPC added to the cellular lipid extracts, the higher the relative amount of NO_2_-POPC (Figure 4A). The interpretation of the MS/MS data acquired using the stepped NCE scheme made it possible to identify the fragment ions formed due to the typical NL of HNO_2_ (Figure 5A). These results are in agreement with those which we described above for the standards of nitrated PCs. These demonstrated that an NCE lower than 25 allowed to observe the typical NL of 47 in the HCD-MS/MS spectrum of NO_2_-POPC (Figure 1A).

This lipidomic approach was also applied to the identification of NO_2_-POPC in GFP-vimentin SW13/cl.2 cells treated with nitrated POPC (Table S13). In this case, to verify the improvement acquired with the optimization of the NCE and to demonstrate the applicability of our methodology that was developed for the identification of nitrated PLs, the DDA was carried out using two different stepped NCE analyses, combining fragment ions with an NCE of 20, 23 and 25 or 25, 30 and 35. The first NCE scheme used the range optimized with the analysis of standards and the second used the range usually applied in our laboratory. The data analysis was performed as previously described, as well as the integration and normalization of the peak area (Table S18). We found the amount of NO_2_-POPC in treated cells to be significantly different from the treated extracts with 1 ng and 8 ng of nitrated POPC (Figure 4A). By using a linear regression model of the normalized peak area of NO_2_-POPC in the treated lipid extracts, we determined that the amount of NO_2_-POPC in the treated cells was equivalent to 3.9 ng (Figure 4B). The HCD-MS/MS spectra obtained for the treated cells (Figure 5B,C) were in the same trend as that observed for the NO_2_-POPC standards (Figure 1): the use of the higher range of NCE led to a decrease in the relative abundance of the product ions formed due to the NL of HNO_2_ (*m*/*z* 758.58). The fragment ions formed due to the NL of HNO_2_ were observed in the HCD-MS/MS acquired with a stepped NCE of 20, 23 and 25 (Figure 5B), but not in the MS/MS data obtained with a stepped NCE of 25, 30 and 35, where the ions at *m*/*z* 310.28, corresponding to the modified fatty acyl chain ((NO_2_-OA − H_2_O + H)^+^), were found (Figure 5C). These results highlight the need to select the most suitable reporter ions according to the experimental conditions, and that selecting the appropriate range of NCE improves the identification of nitro POPC in complex cell lipid extracts.

## 3. Discussion

Nitrated PLs include many structurally diverse lipid species, and its nitro derivatives (NO_2_-PLs) were the main products identified in biological samples, including the H9c2 cardiomyoblasts [10] and cardiac mitochondria of diabetic rats treated with streptozotocin [12]. These modified lipids showed antioxidant properties thanks to their radical scavenging potential against the 2,2’-azino-bis-3-ethylbenzothiazoline-6-sulfonic acid radical cation (ABTS^●+^) and 2,2-diphenyl-1-picrylhydrazyl radical (DPPH^●^) [11,32] and can also act as anti-inflammatory agents by inhibiting the expression of inducible nitric oxide synthase (iNOS; at the protein level) in RAW 264.7 macrophages treated with lipopolysaccharide [32]. Moreover, nitrated POPC induced a series of downstream cellular effects in SW13/cl.2 cells, showing nitrated PLs as new potential electrophilic lipid mediators with selective actions [33]. However, their structural characteristics can have a great impact on their biological significance, as previously reported for NO_2_-FAs (reviewed in [7]). As such, the accurate detection and characterization of these low-abundance modified lipids, in particular by using MS-based lipidomic approaches, are of the utmost importance in helping to understand their potential biological roles.

Lipidomics has been widely applied for the large-scale characterization of lipid profiles and quantification of individual lipid molecular species using either targeted or untargeted MS-based approaches that include shotgun methods and HPLC-MS based strategies. It has also been applied in the study of nitrated PLs by using CID-MS/MS [10,12] and HCD-MS/MS approaches [11,13]. A very recent work on the characterization of nitrated POPS has revealed differences in the fragmentation patterns and reporter ions typical of these modified lipids under both CID- and HCD-MS/MS conditions [11]. The ion activation method and the CE used in tandem MS have been reported as a key step that determines the generation of the structural information observed in the MS/MS spectra of the individual molecular species [20]. It is known that the efficiency of fragmentation of the different molecular species is not equal [34] and should be adjusted to obtain the maximum intensity of the reporter fragment ions [35,36].

In this study, we performed, for the first time, an in-depth characterization of the fragmentation pattern of nitrated PCs and nitrated PEs species under HCD-MS/MS conditions in a Q-Orbitrap mass spectrometer. We assessed the alterations in the fragmentation pattern and the typical reporter ions usually used to identify nitrated PLs, which were previously observed under CID conditions, using nitrated PCs and nitrated PEs generated from *in vitro* model systems, but also in the lipid extracts from cells treated with nitrated POPC in culture (treated cells) and in the lipid extracts treated with nitrated POPC solution before the acquisition of the LC-MS data (treated extracts). We showed that the NCE influenced the intensity of the reporter ions arising from the NL of HNO_2_ (NL 47 Da), which are commonly used for the identification of nitrated and nitroxidized PLs *in vitro* and *in vivo*. Indeed, the intensity of the reporter ions formed due to the typical NL of HNO_2_ was inversely correlated with the increase in NCE. We observed that higher a NCE under HCD-MS/MS conditions was accompanied by a decrease in the relative abundance of the product ions formed due to the typical NL of HNO_2_ and in some cases, hindered the detection of these markers in the MS/MS spectra, preventing their confirmation in the MS/MS analysis. The loss of HNO_2_ was oberved with a relatively high abundance, in the CID-MS/MS of nitrated PCs and nitrated PEs species and until now, it was expected to always be present in all types of tandem mass spectra [10,12]. Our results showed that it was not the case when NO_2_-PLs were characterized by HCD-MS/MS. The absence of this typical fragmentation was recently observed for the characterization of nitrated cardiolipins (CLs) under HCD-MS/MS [13]

In our study, an increase in the intensity yield of other product ions was obtained when the medium (25) and high (30) NCE ranges were applied. Some of these ions were also observed for the CID-MS/MS analysis of nitrated PCs and PEs, namely the ions formed by the combined loss of HNO_2_ and the polar head group [10,12]. Although this seems to be desirable, these results were obtained at the expense of a decrease in the abundance of diagnostic ions. On the other hand, under the HCD-MS/MS conditions, the product ions with the nitrated fatty acyl moiety appeared as the most dominant fragment ions, containing important information on the structural characteristics of nitrated PLs, with particular relevance in the case of the nitrated PE species. These results are in agreement with the previous results reported by Neves and co-authors [11] for nitrated PS derivatives where low *m*/*z* reporter ions, attributed to the carboxylate anions of the modified fatty acyl chains, were seen with a higher relative abundance in the HCD-MS/MS spectra acquired in the orbitrap instrument, while in the CID-MS/MS the product ions with higher *m*/*z* values, as the ones formed by the typical NL of HNO_2_, were the ones seen with a higher relative abundance in MS/MS spectra. Montero-Bullon and co-authors [13] also found that the product ions of nitrated fatty acyl arose as one of the most informative fragmentation pathways of nitrated CLs. These differences are due to the higher energy dissociations in HCD (<100 eV) than those used in the resonant-excitation CID (<2 eV) [37]. Moreover, our results are also in agreement with those reported by Almeida and co-authors [17], who found that the same NCE is not applicable for all lipid species and is also dependent on the lipid class. These results confirmed the need for an evaluation of the NCE to improve the detection of lipid molecular species, in particular those with low abundance. HCD-induced fragmentation can improve the yield of low molecular weight fragment ions carrying important structural information due to the multiple collisions of precursor ions and fragments ions with gas molecules [38]. Moreover, HCD is a higher energy alternative to CID, and therefore a lower NCE is required to achieve the same degree of fragmentation [19,39]. This may be why it is still possible to detect the typical NL of HNO_2_ using a lower NCE.

High-resolution orbitraps have become widely used instruments for MS-based lipidomic approaches. As such, the information on the most appropriate instrument settings and parameters gathered in this study will offer new opportunities to expand the research in the field of the MS analysis of nitrated PLs using orbitrap instruments, allowing the accurate identification of these modified lipid species in complex biological matrices, thus facilitating the elucidation of their pathophysiological roles.

## 4. Materials and Methods

### 4.1. Reagents/Chemicals

The phospholipids standards 1-palmitoyl-2-oleoyl-*sn*-glycero-3-phosphocholine (POPC, C16:0/C18:1), 1-palmitoyl-2-linoleoyl-*sn*-glycero-3-phosphocholine (PLPC, C16:0/C18:2), 1-palmitoyl-2-arachidonoyl-*sn*-glycero-3-phosphocholine (PAPC, C16:0/C20:4) 1-palmitoyl-2-oleoyl-*sn*-glycero-3-phosphoethanolamine (POPE, C16.0/C18:1), 1-palmitoyl-2-linoleoyl-*sn*-glycero-3-phosphoethanolamine (PLPE, C16:0/C18:2), 1-palmitoyl-2-arachidonoyl-*sn*-glycero-3-phosphoethanolamine (PAPE, C16:0/C20:4) and the phospholipid internal standards for the HPLC-MS 1,2-dimyristoyl-*sn*-glycero-3-phosphocholine (dMPC; PC 14:0/14:0) and 1,2-dimyristoyl-*sn*-glycero-3 phosphoethanolamine (dMPE; PE 14:0/14:0) were obtained from Avanti^®^ Polar Lipids, Inc. (Alabaster, AL, USA). These phospholipids had a purity of >99% and were used without further purification. Milli-Q water was used for all the experiments, filtered through a 0.22-µm filter and obtained using a Milli-Q Millipore system (Synergy^®^, Millipore Corporation, Billerica, MA, USA). HPLC grade chloroform, methanol and acetonitrile were purchased from Fisher Scientific Ltd. (Leicestershire, UK). Formic acid, ammonium acetate and solid nitronium tetrafluoroborate (NO_2_BF_4_) were purchased from Sigma-Aldrich (St. Louis, MO, USA). Ammonium molybdate and sodium dihydrogen phosphate dihydrate (NaH_2_PO_4_.2H_2_O) were purchased from Riedel-de Haën (Seelze, Germany). Perchloric acid 70% was purchased from Panreac (Barcelona, Spain). Ascorbic acid was purchased from VWR International (Leicestershire, UK). Penicillin and streptomycin were purchased from Gibco Life Technologies (Paisley, UK). Dulbecco’s modified eagle medium (DMEM) was purchased from Invitrogen (Carlsbad, CA, USA). Fetal bovine serum (FBS) was purchased from Lonza Inc. (Walkersville, MD, USA). Glass bottom dishes were purchased from MatTek Corporation (Ashland, OR, USA). All the chemicals and reagents used were of the highest grade of purity commercially available and were used without further purification.

### 4.2. Nitration of Phospholipids Using In Vitro Model Systems

Phospholipid (PL) standards nitration was carried out with NO_2_BF_4_ as previously described [10,11,12]. A solution of each PL standard (1 mg) in chloroform (1 mL) was prepared in an amber vial tube. Then, an excess of solid NO_2_BF_4_ (~1 mg) was added. The reaction mixture was incubated at room temperature during 1 h and was maintained under orbital shaking at 750 rpm. After incubation, the mixture was transferred to a centrifuge glass tube and the reaction was stopped by solvent extraction with Milli-Q water. The water was added to hydrolyze unreacted NO_2_BF_4_ and/or to separate contaminating anions (such as nitrite (NO_2_^−^), nitrate (NO_3_^−^), or tetrafluoroborate anion (BF_4_^−^) from phospholipids) [40,41]. The mixture was vortexed for 30 s and then centrifuged at 2000 rpm for 10 min at room temperature using a Mixtasel Centrifuge (Selecta, Barcelona, Spain). The organic layer containing the phospholipid products was collected, evaporated under a nitrogen stream and stored at −20 °C to be further quantified by a phosphorous assay [42] and analyzed by direct infusion using a high-resolution mass spectrometer (Q-Exactive hybrid quadrupole Orbitrap^®^).

### 4.3. Phospholipid Quantification by Phosphorous Measurement Assay

The total amount of nitrated PLs recovered after extraction was quantified with the phosphorus assay as previously described by Bartlett and Lewis [42] with modifications. Briefly, 125 µL of concentrated perchloric acid (70% *w*/*v*) was added to an aliquot of 10 µL in each nitrated PC and nitrated PE sample (lipid extracts were dissolved in 1 mL of CHCl_3_; a volume of 10 µL of each sample was dried under a nitrogen stream before the addition of perchloric acid). Samples were then incubated for 1 h at 180 °C in the heating block (Stuart, London, UK). Afterwards, 825 µL of H_2_O, 125 µL of 2.5% ammonium molybdate (*m*/*v*; 2.5 g of NaMoO_4_·H_2_O in 100 mL of H_2_O) and 125 µL of 10% ascorbic acid (*m*/*v*; 0.1 g in 1 mL H_2_O) were added to all the samples, with votex mixing between each addition. Then, the samples were incubated for 10 min at 100 °C in a water bath. The samples were put on cold water afterwards. Standards from 0.1 to 2 μg of phosphorus (sodium dihydrogen phosphate dihydrate (NaH_2_PO_4_.2H_2_O), 100 μg of phosphorus per mL) underwent the same treatment as the samples, except for the heating block phase. Finally, a volume of 200 μL of each standard and sample was added to the 96-multiwell plate and the absorbance was measured at 797 nm in a Multiskan GO Microplate Spectrophotometer (Thermo Fisher Scientific, Waltham, MA, USA). The amount of phosphorus present in each sample was calculated by linear regression through the graph which related the amount of phosphorus present in the standards (X axis) and the absorbance obtained from the duplicates of various concentrations (Y axis). For the lipid extracts, the amount of phospholipid was directly calculated by multiplying the amount of phosphorus obtained by 25, a conversion factor that multiplied the mass of the phosphorus to give the average mass of a phospholipid [43].

### 4.4. HCD-Orbitrap Instrumental Conditions

The high-mass-resolving ESI-MS used for the accurate mass measurements and optimization of the HCD-MS/MS experiment conditions were conducted in a Q-Exactive hybrid quadrupole Orbitrap^®^ mass spectrometer (Thermo Fisher Scientific, Bremen, Germany). The instrument was operated in both positive and negative ion modes, with a spray voltage at 3.0 kV and −2.7 kV, respectively, and interfaced with a HESI II ion source. The samples of the nitrated PC and PE were diluted from 1 mg mL^−1^ stock solutions (in chloroform) using MeOH with 1% (*v*/*v*) formic acid to a final concentration of 1, 2 and 4 µg mL^−1^. The analysis was performed through the direct infusion of the prepared solutions at a flow rate of 12 µL min^−1^ into the ESI source, and the operating conditions were as follows: sheath gas (nitrogen) flow rate 5 (arbitrary units); auxiliary gas (nitrogen) flow rate 1 (arbitrary); capillary temperature 250 °C, and S-lens radio frequency (RF) level 50.

The acquisition method was set with a full scan at a resolution of 70,000; the *m*/*z* range was set to 100–1500 in both the negative and positive ion modes; the automatic gain control (AGC) target was set at 3e6; the maximum injection time (IT) was 250 ms; the capillary temperature was 250 °C; the sheath gas flow was 15 U; the maximum spray current was 100; the probe heater temperature was 50 °C; the S-lens RF level was 50. The full MS spectra were acquired over 20 s in profile mode. The Q Exactive system was tuned and calibrated using the peaks of known mass from a calibration solution (Thermo Scientific) to achieve a mass accuracy of <0.5 ppm RMS (root mean square). The spectra were analyzed using the acquisition software Xcalibur (V3.3, Thermo Scientific, San Jose, CA, USA) [10,11,12].

To obtain the product ion spectra (HCD-MS/MS) of the nitrated derivatives of POPC, PLPC, PAPC, POPE, PLPE and PAPE during the ESI-MS experiments, the selected precursor ions were isolated by the quadrupole and sent to the HCD cell for fragmentation via the C-trap. In the MS/MS mode, the mass resolution of the Orbitrap analyzer was set at 70,000; the AGC target was set at 3e6; the maximum IT was 250 ms; the isolation window was of 1.0 *m*/*z*; the MS/MS spectra were acquired in centroid mode; and three different normalized collision energies (NCEs) were used: 20, 25 and 30 (arbitrary units). Nitrogen was also used as the collision gas. Each MS/MS spectra were acquired over 20 s. All experiments were repeated three times.

### 4.5. Cell Culture and Treatments of Adrenal Carcinoma SW13/cl.2 Cells

The cell line SW13/cl.2 (SW13) was stably transfected with an expression plasmid coding for GFP-vimentin wild type (wt) as previously described by Peréz-Sala and co-authors [44]. In these cells, GFP-vimentin constructs formed an extended meshwork of squiggles and short filaments. The GFP-vimentin wt SW13/cl.2 cells were cultured in p-60 plates in Dulbecco’s modified eagle medium (DMEM) supplemented with 10% (*v*/*v*) fetal bovine serum (FBS) and 1% antibiotics (100 U mL^−1^ penicillin and 100 µg mL^−1^ streptomycin) at 37 °C under 5% CO_2_. The experiments were performed on confluent monolayers in a serum-free medium. The cells were treated with 10 µmol L^−1^ nitrated POPC (from 3.67 mmol/L stock solution in DMSO) for 6 h. The control cells received an equivalent volume of vehicle (DMSO), as required. At the time point, the cells were collected by scraping in phosphate buffered saline (PBS) on ice and centrifuged at 1000 rpm for 5 min. The cell pellets were stored at −80 °C until used [33]. All experiments were repeated three times.

### 4.6. Lipid Extraction from SW13/cl.2 Cells

The lipid extraction of both the untreated (control cells) and the nitrated POPC-treated SW13/cl.2 cells was performed according to the method of Bligh and Dyer [45], with modifications [14]. Briefly, 3.75 mL chloroform:methanol 1:2 (*v*/*v*) was added to each cell pellet previously resuspended in 1 mL of Milli-Q H_2_O. The samples were well vortexed and incubated on ice for 30 min. An additional volume of 1.25 mL of chloroform and 1.25 mL of Milli-Q H_2_O were added followed by vortexing for 1 min between each addition. The samples were centrifuged at 1000 rpm for 5 min at room temperature using a Mixtasel Centrifuge (Selecta) to obtain a two-phase system: an aqueous top phase and an organic bottom phase. The lipid extract was recovered from the organic bottom phase. In order to guarantee a full extraction, a volume of 1.88 mL of chloroform was added to the remaining aqueous phase followed by vortexing and new centrifugation. The organic phase was recovered to the same tube as before and dried under a nitrogen stream. After drying, the total lipid extracts were resuspended in 300 µL of chloroform and transferred to an amber glass vial. This step was repeated twice to ensure the full recovery of the lipid extracts. The phospholipid amount in each lipid extract was determined by the phosphorous measurement performed according to Bartlett and Lewis, as previously described [10,11,12,42].

### 4.7. HPLC-ESI-MS and MS/MS Analysis

The lipid extracts from the SW13/cl.2 cells (untreated and treated with 10 µmol L^−1^ of nitrated POPC during the culture phase) were separated by hydrophilic interaction liquid chromatography (HILIC-LC-MS) using a high performance-LC (HPLC) system (Ultimate 3000 Dionex, Thermo Fisher Scientific, Bremen, Germany) with an autosampler (sampler chamber temperature was set at 5 °C) and a 20 µL sample loop. The HPLC system was coupled online to the Q-Exactive^®^ hybrid quadrupole Orbitrap^®^ mass spectrometer (Thermo Fisher Scientific, Bremen, Germany) [14]. The solvent system consisted of two mobile phases as follows: mobile phase A (acetonitrile:methanol:water 50:25:25 (*v*/*v*/*v*) with 1 mM ammonium acetate) and mobile phase B (acetonitrile:methanol 60:40 (*v*/*v*) with 1 mM ammonium acetate). Initially, 40% of mobile phase A was held isocratically for 8 min, followed by a linear increase to 60% of A within 7 min and a maintenance period of 5 min, returning to the initial conditions in 15 min (5 min to decrease to 40% of A and a re-equilibration period of 10 min prior to the next injection). For the untreated cells (control cells), a volume of 5 µL of each sample containing 5 µg of phospholipid extract, 4 µL of phospholipid standards mix (dMPC—0.02 µg and dMPE—0.02 µg), 1, 2, 4 and 8 ng of nitrated POPC (1µg mL^−1^) and an appropriate volume of eluent B (final volume 100 µL) was introduced into the Ascentis^®^ Si column (15 cm × 1 mm, 3 µm, Sigma-Aldrich) with a flow rate of 40 µL min^−1^ and at 30 °C. These samples were labelled as treated extracts. For the nitrated POPC-treated cells, a volume of 5 µL containing 5 µg of phospholipid extracts, 4 µL of phospholipid standards mix (dMPC—0.02 µg and dMPE—0.02 µg) and an 91 µL of eluent B (final volume of 100 µL) was used. These samples were labelled as the treated cells. The mass spectrometer with Orbitrap^®^ technology operated simultaneously in the positive (electrospray voltage 3.0 kV) and negative (electrospray voltage −2.7 kV) modes with a high resolution of 70,000 and a AGC target of 1e6, the capillary temperature was 250 °C, the sheath gas flow was 15 U, the auxiliary gas flow was 5 U, the maximum spray current was 100, the probe heater temperature was 130 °C, the S-lens RF level was 50, the maximum injection time was 100 ms and the scan range was *m*/*z* 200–1600 *m*/*z* (profile). In the MS/MS experiments, a resolution of 17,500, an AGC target of 1e5, a maximum injection time of 50 ms, the scan range of *m*/*z* 200–2000 *m*/*z* (centroid) and an isolation width of 1.0 *m*/*z* were used and the cycles consisted of one full scan mass spectrum and ten data-dependent MS/MS scans that were repeated continuously throughout the experiments, with the dynamic exclusion of 60 s and the intensity threshold of 2e4. The samples were analysed using stepped energy which effectively combined the fragmentation at energies of 20, 23 and 25 or 25, 30 and 35. The data acquisition was carried out using the Xcalibur data system (V3.3, Thermo Fisher Scientific, Waltham, MA, USA). The experiments were performed using three biological replicates, acquired on two different days.

### 4.8. Data and Statistical Analysis

The peak area integration and assignments of NO_2_-POPC and minor dinitro and nitroxidized species were performed using MzMine 2.42 [31]. The software allowed for a filtering, a peak detection ((M + H)^+^ ions), peak processing, peak alignment, and an assignment against an in-house made database. A *m*/*z* tolerance of +/− 5 ppm and a retention time (RT) tolerance of +/− 2 min was used. The validation of all assignments was made by a manual analysis of the MS/MS data, whenever it was possible. For the relative quantitation, we used the Excel tool (Excel, Microsoft, Redmond, WA, USA) to analyse the exported integrated peak area values. A statistical analysis was performed using one-way analysis of variance (ANOVA) and Tukey’s multiple comparison tests in PRISM^®^ GraphPad Software, Inc (GraphPad Prism 5.0, La Jolla, CA, USA).

## 5. Conclusions

In MS/MS experiments using Orbitrap mass spectrometers, the relative abundance of the reporter ions of nitrated and nitroxidized PLs is significantly affected by the range of NCE used. This will determine which fragment ions are observed and the success of the detection of these modififed lipids in biological samples. The fine-tuning of an NCE value under the MS/MS experiments is necessary and extremely important to maximize the identification of nitrated and nitroxidized PLs by the generation of reporter ions with sufficient intensity to guarantee their accurate assignment.

Our results underline the importance of improving the methodology to obtain informative data in which MS/MS spectra match the typical fragmentation pattern of target compounds and allow to identify typical reporter ions. We believe that the accurate identification of these modified PLs will contribute to the understanding of their roles and signalling pathways.

## Figures and Tables

**Table 1:** Summary of the main fragmentation pathways observed in the higher-energy collision dissociation (HCD)- tandem mass spectrometry (MS/MS) spectra of the (M + H)^+^ ions of nitro (NO_2)_ phospholipids (PLs) (NO_2_-PLs) formed after the reaction of phosphatidylcholines (PCs) and phosphatidylethanolamines (PEs) with nitronium tetrafluoroborate (NO_2_BF_4_), with the proposed identification and *m*/*z* values: 1-palmitoyl-2-oleoyl-*sn*-glycero-3-phosphocholine (POPC), PC16:0/18:1; 1-palmitoyl-2-linoleoyl-*sn*-glycero-3-phosphocholine (PLPC), PC16:0/18:2; 1-palmitoyl-2-arachidonoyl-*sn*-glycero-3-phosphocholine (PAPC), PC16:0/20:4 and 1-palmitoyl-2-oleoyl-*sn*-glycero-3-phosphoethanolamine (POPE), PE16:0/18:1; 1-palmitoyl-2-linoleoyl-*sn*-glycero-3-phosphoethanolamine (PLPE), PE16:0/18:2; 1-palmitoyl-2-arachidonoyl-*sn*-glycero-3-phosphoethanolamine (PAPE), PE16:0/20:4.

**Typical Neutral Losses and Product Ions Observed for NO_2_-PC and NO_2_-PE in Positive Ion Mode**							
**Neutral Losses**	**Proposed Identification**	**(POPC + NO_2_ + H)^+^**	**(POPE + NO_2_ + H)^+^**	**(PLPC + NO_2_ + H)^+^**	**(PLPE + NO_2_ + H)^+^**	**(PAPC + NO_2_ + H)^+^**	**(PAPE + NO_2_ + H)^+^**
		805.8	763.6	803.6	761.5	827.8	785.6
18 Da	-H_2_O	787.6	745.5	785.5	743.5	809.6	767.5
43 Da	-C_2_H_5_N	---	720.4	--	718.4	--	742.5
47 Da	-HNO_2_	758.7	716.5	756.5	714.6	780.6	738.5
59 Da	-C_3_H_9_N	746.5	--	744.34	--	768.7	--
90 Da (43 + 47)	-(C_2_H_5_N + HNO_2_)	--	673.4	--	671.6	--	695.5
106 Da (59 + 47)	-(C_3_H_9_N + HNO_2_)	699.6	---	697.4	--	721.5	--
141 Da	-C_2_H_8_PO_4_N	--	622.5	--	620.5	--	644.5
183 Da	-C_5_H_14_PO_4_N	622.6	--	620.6	--	644.6	--
188 Da (141 + 47)	-(C_2_H_8_PO_4_N + HNO_2_)	---	575.4	--	573.4	--	597.6
230 Da (183 + 47)	-(C_5_H_14_PO_4_N + HNO_2_)	575.5	--	573.6	--	597.7	--
238 Da	-R_1_C=C=O	567.6	525.4	565.4	523.3	589.3	547.4
256 Da	-R_1_COOH	549.6	507.4	547.4	505.5	571.5	529.5
**Product Ions**							
		**Nitro Oleic Acid (NO_2_-OA)**		**Nitro Linoleic Acid (NO_2_-LA)**		**Nitro Arachidonic Acid (NO_2_-AA)**	
(NO_2_-FA + H)^+^		328.3	328.3	326.4	326.4	350.3	350.3
(NO_2_-FA-H_2_O + H)^+^		310.3	310.3	308.3	308.3	332.3	332.3
(NO_2_-FA-2H_2_O + H)^+^		292.3	292.3	290.3	290.3	314.2	314.2
(NO_2_-FA-NO_2_ + H)^+^		281.3	281.3	279.3	279.3	303.3	303.3

## Supplementary Materials

Supplementary Materials are available online at https://www.mdpi.com/1420-3049/25/9/2120/s1.
