# Supplementary material for: Advancing Target Identification of Nitrated Phospholipids in Biological Systems by HCD Specific Fragmentation Fingerprinting in Orbitrap Platforms
Source: Molecules. 2020 May 1;25(9):2120. doi: 10.3390/molecules25092120 (PMC7248851; doi:10.3390/molecules25092120)
Supplement: Supplementary file 1 [file molecules-25-02120-s001.zip › Supplementary Material_Molecules_Neves et al_2020.doc]

Article

Advancing target identification of nitrated phospholipids in biological systems by HCD specific fragmentation fingerprinting in Orbitrap platforms

**Bruna Neves^1,2^, Sofia Duarte^3^, Pedro Domingues^1^, Dolores Pérez-Sala^3^, Maria Manuel Oliveira^4^, Maria do Rosário Domingues^1,2^ and Tânia Melo^1,2*^**

^1^	Mass Spectrometry Centre, LAQV-REQUIMTE, Department of Chemistry, University of Aveiro, Campus Universitário de Santiago, 3810-193 Aveiro, Portugal; [brunabneves@ua.pt](mailto:brunabneves@ua.pt) (BN); [p.domingues@ua.pt](mailto:p.domingues@ua.pt) (PD); [mrd@ua.pt](mailto:mrd@ua.pt) (MRD); [taniamelo@ua.pt](mailto:taniamelo@ua.pt) (TM)

^2^	Centre for Environmental and Marine Studies, CESAM, ECOMARE, Department of Chemistry, University of Aveiro, Campus Universitário de Santiago 3810-193 Aveiro, Portugal; [brunabneves@ua.pt](mailto:brunabneves@ua.pt) (BN); [mrd@ua.pt](mailto:mrd@ua.pt) (MRD); [taniamelo@ua.pt](mailto:taniamelo@ua.pt) (TM)

^3^	Department of Structural and Chemical Biology, Centro de Investigaciones Biológicas Margarita Salas, CSIC, 28040 Madrid, Spain; sofiaineslealduarte@gmail.com (SD); dperezsala@cib.csic.es (DPS)

^4^	Department of Chemistry, University of Trás-os-Montes e Alto Douro, 5000-801 Vila Real, Portugal; [mmso@utad.pt](mailto:mmso@utad.pt) (MMO)

*****	Correspondence: [taniamelo@ua.pt](mailto:taniamelo@ua.pt); Tel.: +351 234 370 200

**Figure S1:** **(A)** HCD-MS/MS spectra of [NO_2_-POPC+H]^+^ ions at *m/z* 805.5 obtained at low (20), medium (25), and high (30) NCE for NO_2_-POPC. Neutral loss of HNO_2_ can be observed at *m/z* 758.5. **(B)** Effect of low (20), medium (25), and high (30) NCE in the intensity of the reporter ions of nitro POPC (NO_2_-POPC, *m/z* 805.5) observed in positive ion mode that corresponds to the neutral loss of nitro group (HNO_2_, *m/z* 758.5).

**Figure S2:** **(A)** HCD-MS/MS spectra of [NO_2_-POPE+H]^+^ ions at *m/z* 763.5 obtained at low (20), medium (25), and (30) NCE for NO_2_-POPE. Neutral loss of HNO_2_ can be observed at *m/z* 716.5. **(B)** Effect of low (20), medium (25), and high (30) NCE in the intensity of the reporter ions of nitrated POPE (NO_2_-POPE, *m/z* 763.5) observed in positive ion mode that corresponds to the neutral loss of nitro group (HNO_2_, *m/z* 716.5).

**Figure S3: (A)** HCD-MS/MS spectra of [NO_2_-POPE−H]^−^ ions at *m/z* 761.5 obtained at low (20), medium (25), and (30) NCE for NO_2_-POPE. Neutral loss of HNO_2_ can be observed at *m/z* 714.5. **(B)** Effect of low (20), medium (25), and high (30) NCE in the intensity of the reporter ions of nitrated POPE (NO_2_-POPE, *m/z* 761.5) observed in negative ion mode that corresponds to the neutral loss of nitro group (HNO_2_, *m/z* 714.5).

**Figure S4:** Effect of low (20), medium (25) and high (30) NCE in the intensity of the fragment ions with lower *m/z* values observed in the HCD-MS/MS spectra of NO_2_-POPC in positive ion mode (from the [M+H]^+^ ions, **A**) and of NO_2_-POPE both in positive (from the [M+H]^+^ ions, **B)** and negative ion mode (from the [M−H]^−^ ions, C) obtained when using three concentrations of NO_2_-PL (1 µg mL^−1^, 2 µg mL^−1^ and 4 µg mL^−1^). Fragment ions at *m/z* 575.5 correspond to the combined neutral loss of HNO_2_ with phosphocholine (NL 47 plus 183 Da) or phosphoethanolamine polar heads (NL 47 plus 141 Da). Fragment ions at *m/z* 496.3 and 478.3 **(A)** and at *m/z* 454.3 and 436.3 **(B)** correspond to the neutral loss of nitrated oleic acid (NO_2_-OA) as keto (NL of 309 Da, (NO_2_-OA−H_2_O)) and acid derivatives (NL of 327 Da, NO_2_-OA), respectively. Fragment ions at *m/z* 328.2 and 310.2 (**A** and **B**) correspond to the [NO_2_-OA+H]^+^ and [NO_2_-OA−H_2_O+H]^+^ ions, respectively. Fragment ions at *m/z* 452.3 and 434.3 **(C)** correspond to the neutral loss of nitrated oleic acid (NO_2_-OA) as keto and acid derivative, respectively, and fragment ions at *m/z* 326.3 and 308.3 correspond to the [NO_2_-OA−H]^−^ and [NO_2_-OA-H_2_O−H]^−^ ions, respectively.
